# Effectiveness of Electronic Guidelines (GERH^®^) to Improve the Clinical Use of Antibiotics in An Intensive Care Unit

**DOI:** 10.3390/antibiotics9080521

**Published:** 2020-08-15

**Authors:** Paola Navarro-Gómez, Jose Gutierrez-Fernandez, Manuel Angel Rodriguez-Maresca, Maria Carmen Olvera-Porcel, Antonio Sorlozano-Puerto

**Affiliations:** 1 Laboratory Clinical Management Unit, Torrecardenas Hospital Complex, 04009 Almeria, Spain; paola.ng24@gmail.com (P.N.-G.); manuel.rodriguez.maresca.sspa@juntadeandalucia.es (M.A.R.-M.); 2 Department of Microbiology, School of Medicine and PhD Program in Clinical Medicine and Public Health, University of Granada-ibs, 18016 Granada, Spain; asp@ugr.es; 3 Andalusian Public Foundation for biomedical research in eastern Andalusia, Alejandro Otero-FIBAO, Torrecardenas Hospital Complex, 04009 Almeria, Spain; molvera@fibao.es

**Keywords:** intensive care unit, computerized clinical decision support system, antibiotics

## Abstract

The objective of the study was to evaluate the capacity of GERH^®^-derived local resistance maps (LRMs) to predict antibiotic susceptibility profiles and recommend the appropriate empirical treatment for ICU patients with nosocomial infection. Data gathered between 2007 and 2016 were retrospectively studied to compare susceptibility information from antibiograms of microorganisms isolated in blood cultures, lower respiratory tract samples, and urine samples from all ICU patients meeting clinical criteria for infection with the susceptibility mapped by LRMs for these bacterial species. Susceptibility described by LRMs was concordant with in vitro study results in 73.9% of cases. The LRM-predicted outcome agreed with the antibiogram result in >90% of cases infected with the bacteria for which GERH^®^ offers data on susceptibility to daptomycin, vancomycin, teicoplanin, linezolid, and rifampicin. Full adherence to LRM recommendations would have improved the percentage adequacy of empirical prescriptions by 2.2% for lower respiratory tract infections (*p* = 0.018), 3.1% for bacteremia (*p* = 0.07), and 5.3% for urinary tract infections (*p* = 0.142). LRMs may moderately improve the adequacy of empirical antibiotic therapy, especially for lower respiratory tract infections. LRMs recommend appropriate prescriptions in approximately 50% of cases but are less useful in patients with bacteremia or urinary tract infection.

## 1. Introduction

Healthcare-associated infections are those developed by patients as result of care received in hospital (known as nosocomial infections) or any other healthcare setting [1]. Nosocomial infections are those that appear at least 72 h after hospital admission and were not previously present or incubating [2,3]. They indicate the care quality delivered by hospitals and are related to increased morbidity and mortality and a longer hospital stay, representing an important public health problem and increasing healthcare costs [4]. Nosocomial infections are estimated to affect 5–10% of patients admitted to hospital [5], although their prevalence varies among departments and hospitals, being three- to five-fold higher in intensive care units (ICUs) than in other hospital areas [6,7]. They are considered to be the sixth cause of death in Europe and the USA, but around one-third of them could be prevented by infection control programs and adequate hygiene measures [8]. The magnitude of the attributable mortality is controversial and depends on the type of infection, the severity of the patient (APACHE II scale), and the length of hospital stay, among many other factors [9,10]. However, the earliest possible prescription of an appropriate empirical antibiotic treatment of nosocomial infections is known to be a key factor to improve the survival of ICU patients [11].

In ICU patients, nosocomial infections are often produced by multi-resistant microorganisms [12], complicating prescribing decisions. The scant development of new active ingredients has prompted novel strategies to extend the usefulness of existing antibiotics against severe infections [13], including the local study of bacterial resistance phenotypes. This allows a more precise selection of antibiotics based on local knowledge of the microorganisms most frequently responsible for infection in each hospital area [14].

Since 2012, Spanish hospitals have implemented programs to optimize the use of antibiotics (*Programas de Optimización del Uso de Antimicrobianos* (PROA)). The main objectives are to improve the clinical outcomes of patients with infections; minimize antimicrobial-related adverse effects, including the development and spread of resistance; and promote more effective treatments with lower health costs. PROA recommendations include accessibility to microbiological data, knowledge of bacterial resistance percentages (especially in the hospital area), and the implementation of measures to assist prescribing decisions. Computerized clinical decision support systems have proven useful to meet these objectives [13].

PROA implementation in our hospital (Torrecardenas Hospital Complex, Almeria, Spain) led to the development in 2014 of a computerized clinical decision support program for the prescription of antimicrobials, named in Spanish the *Guía Electrónica de Resistencias Hospitalarias* (GERH^®^). Using this system, updated identification and susceptibility data from microbiological studies in all hospitalized patients can be rapidly communicated to physicians using a secure hospital intranet system. Two GERH^®^-based applications were launched: local resistance maps (LRMs) and preliminary microbiological reports with therapeutic recommendations (PMRTRs). To date, these applications have only been available to ICU physicians. 

The objective of this study was to evaluate the capacity of LRMs to predict antibiotic susceptibility and resistance profiles and improve the appropriateness of empirical treatments for ICU patients with nosocomial infections, including bacteremia and/or lower respiratory or urinary tract infections.

## 2. Methods 

GERH^®^ is based on the Microsoft.NET Framework with Visual C# and SQL and Open Database Connectivity (ODBC) to the laboratory information system (*Sistema de Información de Laboratorio* (SIL)) of the hospital microbiology laboratory. It is installed on a central server and has been used by ICU physicians since 2014 to consult all susceptibility results stored in the SIL since 2006. The data are organized according to the hospital department, date/date interval, sample type, microorganism/s isolated, and antimicrobials tested, and graphs are created for the ready visualization and interpretation of these data [15].

### 2.1. Local Resistance Maps (LRMs)

**Figure 1:**
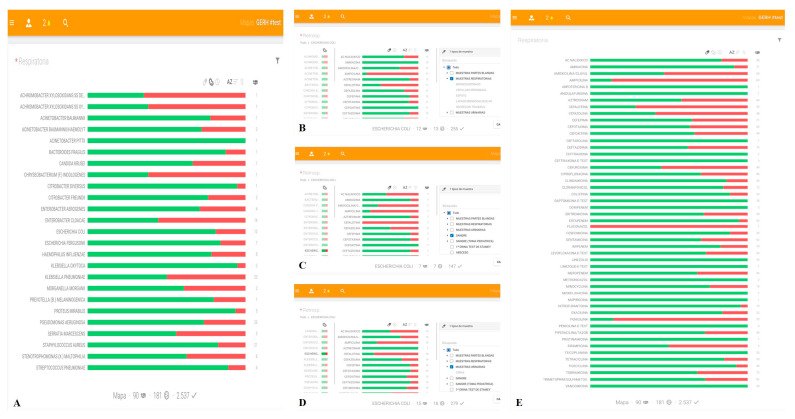
GERH^®^-derived local resistance maps (LRMs). The search criteria in LRMs offer different types of graph. As an example, this figure depicts the following: (**A**) graph showing the frequency of isolation of each bacterial species in ICU patients with lower respiratory tract infection between 1 January and 31 December 2015 alongside the accumulated susceptibility; (**B**) graph showing the accumulated susceptibility of *E. coli* in isolates obtained in respiratory samples (bronchial aspirate, bronchial brushing, sputum, bronchoalveolar lavage or tracheal secretion) of ICU patients between 1 January and 31 December 2015; (**C**) graph showing the accumulated susceptibility of *E. coli* in isolates obtained in blood samples of ICU patients between 1 January and 31 December 2015; (**D**) graph showing the accumulated susceptibility of *E. coli* in isolates obtained from urine samples of ICU patients between 1 January and 31 December 2015; and (**E**) graph showing the activity of various antimicrobials, considering the group of microorganisms isolated in respiratory samples of ICU patients between 1 January and 31 December 2015, in which green indicates the percentage of microorganisms of the total tested in which each antibiotic was active. In (**A**–**D**), the percentage of susceptible bacteria is represented using a color code: green for the percentage of susceptible isolates and red for the percentage of isolates with intermediate or resistant clinical category.

LRMs (Figure 1) graphically depict information on the frequency of isolated microorganisms (Figure 1A), bacterial susceptibility (Figure 1B–D), and antibiotic activity (Figure 1E). The physicians select and access the graphs via touch screens connected to the hospital intranet. Data are automatically updated every 24 h to include new records from the central GERH^®^ server [15].

The LRMs were derived from analyses of the outcomes obtained in all in vitro susceptibility assays for bacteria isolated in ICU patients with bacteremia, lower respiratory tract infection, or urinary tract infection within a defined time interval, commonly the 12-month period before the consultation. The graphs depict accumulated information for the selected time period on the antibiotic susceptibility profile of isolated bacteria, indicating the likelihood that the infection in question is caused by specific bacteria as well as the expected activity of antibiotics against them. This allows the ICU physician to make informed decisions about the treatment of cases based on the local bacterial epidemiology and on the predicted susceptibility profile of the bacteria isolated. 

This instrument followed the recommendation of the Spanish Society of Infectious Diseases and Clinical Microbiology (Spanish abbreviation: SEIMC) for accumulated reports on antimicrobial susceptibility to include solely microorganisms obtained from human clinical samples with susceptibility results verified by clinical microbiologists. When the same microorganisms are isolated multiple times in the same patient, those with a change in their resistance phenotype to one or more antibiotics are considered [16].

### 2.2. Study Design

This retrospective study compared the concordance obtained each year from 2007 to 2016 between susceptibility data from the antibiograms of microorganisms isolated in blood cultures, respiratory samples (bronchial aspirate, bronchial brushing, sputum, bronchoalveolar lavage, and/or tracheal secretion), and/or urine samples from all ICU patients meeting clinical criteria for infection and the susceptibility data depicted by LRMs for the same bacterial species, based on accumulated data for the whole year. In the LRMs, bacteria were defined as susceptible to an antibiotic when at least 75% of clinical isolates of this bacterial species were susceptible according to in vitro tests [15]. Concordance was defined as agreement between the susceptibility evaluated by LRMs and the susceptibility obtained in the in vitro study in the microbiology laboratory, i.e. when bacteria were considered as susceptible or resistant by both methods, including “intermediate resistance” within the “resistant” category. There was no concordance when the bacteria were considered susceptible by one approach and resistant by the other. Duplicate bacteria with the same identification and antibiogram were excluded, whether obtained from the same sample or isolated in multiple samples from the same patient.

The adequacy of empirical antibiotic treatments was also retrospectively studied, considering them appropriate when active against the bacterial pathogen causing the infection [13]. Accordingly, we determined whether each antibiotic prescribed was active against the bacteria isolated in the different clinical samples from each patient, based on the antibiogram data. The percentage adequacy of empirical prescriptions if they had been based on LRM recommendations was calculated as the number of times that LRM results for the susceptibility of a bacterium to each antibiotic agreed with the result of the susceptibility study as a percentage of the total number of isolates tested. The adequacy of the actual empirical treatment prescribed by physicians was evaluated with reference to the activity of the antibiotic(s) against each bacterium isolated in the different clinical samples according to the corresponding antibiograms. Data on the antibiotics prescribed in ICU patients were retrospectively obtained from the Spanish national nosocomial infection surveillance program (Spanish abbreviation: ENVIN).

Although the instrument is available in the ICU, therapeutic decisions do not have to be based on the data it provides. For this reason, we did not consider or gather data on the compliance of prescribed treatments with LRM recommendations.

### 2.3. Bacteria Selection 

Study inclusion criteria for bacteria were: (i) isolation in blood, respiratory, or urine samples; (ii) definitive microbiological species identification; and (iii) belonging to one of the following bacterial groups: (a) *Enterobacteriaceae* (*Citrobacter* spp., *Enterobacter* spp., *Escherichia* spp., *Klebsiella* spp., *Morganella* spp., *Proteus* spp., *Providencia* spp., or *Serratia* spp.); (b) non-fermenting Gram-negative bacilli (*Acinetobacter* spp., *Pseudomonas* spp., and *Stenotrophomonas* spp.)*;* (c) coagulase-positive staphylococci (*Staphylococcus aureus*), and coagulase-negative staphylococci (*Staphylococcus auricularis, Staphylococcus capitis, Staphylococcus epidermidis, Staphylococcus haemolyticus, Staphylococcus hominis, Staphylococcus intermedius, Staphylococcus saprophyticus, Staphylococcus simulans*, and *Staphylococcus warneri*); (d) *Streptococcus pneumoniae*; (e) enterococci (*Enterococcus faecalis* and *Enterococcus faecium*); or (f) *Haemophilus* spp.

### 2.4. Antibiotic Selection

The following antibiotics were included in relation to the above bacteria: (a) amikacin, amoxicillin-clavulanic acid, aztreonam, cefepime, cefotaxime, ceftazidime, ceftriaxone, cefuroxime, ciprofloxacin/levofloxacin, gentamicin, imipenem, meropenem, piperacillin-tazobactam, and tobramycin in *Enterobacteriaceae*; (b) amikacin, cefepime, ceftazidime, ciprofloxacin/levofloxacin, colistin, gentamicin, imipenem, meropenem, piperacillin-tazobactam, and tobramycin in non-fermenting Gram-negative bacilli; (c) ciprofloxacin/levofloxacin, clindamycin, daptomycin, erythromycin, gentamicin, linezolid, oxacillin, rifampicin, teicoplanin, tobramycin, and vancomycin in staphylococci; (d) cefotaxime, levofloxacin, linezolid, and penicillin in *S. pneumoniae*; (e) ampicillin, levofloxacin, daptomycin, linezolid, teicoplanin, and vancomycin in enterococci; and (f) amoxicillin-clavulanic acid, ampicillin, cefotaxime, ciprofloxacin/levofloxacin, and erythromycin in *Haemophilus* spp.

### 2.5. Study Variables 

Data gathered from antibiogram results, LRMs, and the ENVIN platform were: type of infection, date of sample gathering, type of sample (blood, respiratory, or urine), bacteria identified in each sample, antibiogram of the microorganism(s) isolated, concordance between antibiogram and LRM data, empirical antibiotic treatment prescribed, and concordance with the antibiogram result.

### 2.6. Statistical analysis 

In the statistical analysis, the chi-square test was used to compare the adequacy of the actual empirical treatment with the adequacy of the LRM-recommended treatment, considering *p* < 0.05 to be significant. 

## 3. Results

### 3.1. Concordance Between LRMs and Susceptibility In Vitro

Table 1 compares the susceptibility data for each bacterium and antibiotic according to in vitro studies with those provided by LRMs after analyzing the information accumulated during the previous year. During the study period (2007–2016), the results of 22,520 in vitro trials were compared to the LRM data, obtaining an average concordance of 73.9%. In other words, the susceptibility or resistance described by LRMs for each bacterium–antibiotic association agreed with the in vitro study results in 73.9% of cases.

For enterobacteria, the concordance ranged from a mean of 95.9% for amikacin over the study period (range 89.7–100%) to mean of 63.6% (range 47.6–86.7%) for ciprofloxacin/levofloxacin. In other words, when an enterobacterium was isolated, the expected susceptibility outcome was the same according to both the LRM and antibiogram in 95.9% of cases for amikacin and in 63.6% of cases for ciprofloxacin/levofloxacin. An intermediate degree of concordance was obtained for the other antibiotics studied. 

For non-fermenting Gram-negative bacilli, the concordance widely varied among different antibiotics, obtaining the highest percentage agreement for colistin (88.3%; range 87.5–97.3%) and tobramycin (86.2%; range 58.2–100%) and lower degrees of concordance for amikacin (76.3%; range 42.9–100%), cefepime (54.9%; range 31.3–86.4%), ceftazidime (53.1%; range 28.2–68.8%), ciprofloxacin/levofloxacin (68.8%; range 43.2–88.3%), gentamicin (62.1%; range 34.3–85.0%), piperacillin-tazobactam (60.6%; range 20.0–96.6%), imipenem (49.4%; range 32.4–62.3%), and meropenem (50,7%; range 36.0–71.4%).

For staphylococci, the highest concordance was obtained for daptomycin (99.7%; range 99.0–100%), followed by vancomycin (98.7%; range 95.2–100%), linezolid (92.9%; range 79.4–100%), teicoplanin (92.2%; range 82.1–100%), and rifampicin (90.9%; range 53.6–99.0%). A lower percentage agreement was found for ciprofloxacin/levofloxacin (64.6%; range 54.8–88.6%), clindamycin (60.2%; range 39.6–79.7%), erythromycin (71.0%; range 63,9–84.1%), gentamicin (68.1%; range 53.4–83.1%), oxacillin (71.9%; range 59.5–91.7%), and tobramycin (59.1%; range 50.5–69.7%).

For the remaining bacteria under study (*S. pneumoniae*, enterococci and *Haemophilus* spp.), there were few comparative data and the mean percentage agreement was generally high but showed a very wide range.

Considering all bacteria and antibiotics included in the 22,520 comparisons conducted during the study period, the highest percentage concordance between LRMs and antibiograms was observed for daptomycin (99.7%; range 99.0–100%), vancomycin (98.7%; range 95.5–100%), teicoplanin (92.5%; range 83.0–100%), linezolid (92.3%; range 79.3–100%), and rifampicin (90.9%; range 53.6–99.0%). In summary, the susceptibility data offered by LRMs for bacteria on which the GERH^®^ has this information agrees with the antibiogram result in >90% of cases. 

Table 2 compares the in vitro and LRM susceptibility data for each bacterium and antibiotic by year and by infection type. The mean percentage concordance was 73.5% in lower respiratory tract infections (range 66.5–80.0%), 69.3% in urinary tract infections (range 54.7–81.6%), and 76.1% in bacteremia (range 68.9–80.8%). These findings indicate that the susceptibility information provided by LRMs for these infections is in agreement with the actual susceptibility of the isolated bacteria in 73.5%, 69.3%, and 76.1% of cases, respectively.

### 3.2. Adequacy of Actual Empirical Prescription and Susceptibility Obtained in the Antibiogram 

Table 3 displays the percentage adequacy of LRM-recommended empirical prescriptions for bacteria in relation to the actual susceptibility observed for them. Antibiotics with a percentage adequacy >80% in the empirical antibiotic prescription were amikacin, colistin, daptomycin, linezolid, teicoplanin, and vancomycin. Thus, in relation to amikacin, out of 981 isolates of enterobacteria or non-fermenting Gram-negative bacilli isolated in samples, 904 were susceptible to this antibiotic and 77 were resistant, while its use was recommended by LRMs in 854 of cases, giving a percentage adequacy of 87.1%. Accordingly, if amikacin had been used as empirical treatment when recommended by LRMs, this treatment would have been appropriate in 87.1% of cases in which enterobacteria or non-fermenting Gram-negative bacilli were isolated. For daptomycin, linezolid, teicoplanin, and vancomycin the LRM-recommended treatment would have been appropriate in 99.7%, 92.0%, 92.2%, and 98.7% of cases in which a Gram-positive coccus (staphylococcus, enterococcus, or pneumococcus) was isolated.

Table 4 exhibits the percentage adequacy of empirical antibiotic prescriptions if they had followed LRM recommendations, being 57.6% for lower tract respiratory infections, 41.4% for bacteremia, and 54.9% for urinary tract infections. Table 5 lists the percentage adequacy of the empirical antibiotics actually prescribed, being 55.4% for lower respiratory tract infections, 38.3% for bacteremia, and 49.6% for urinary tract infections. Hence, if LRM recommendations had always been followed in the ICU, the percentage adequacy of prescriptions would have been improved by 2.2% for lower respiratory tract infections (57.6% vs. 55.4%; *p* = 0.018), 3.1% for bacteremia (41.4% vs. 38.3%; *p* = 0.070), and 5.3% for urinary tract infections (54.9% vs. 49.6%; *p* = 0.142).

## 4. Discussion 

A major factor in the emergence of bacterial resistances is the inappropriate prescription of antibiotics [17], estimated to represent 30–50% of all antibiotic prescriptions [18]. For this reason, analysis of the antibiotic susceptibility of microorganisms is not only of major epidemiological and clinical importance but provides invaluable support for prescribing decisions. The use of computerized systems based on laboratory susceptibility results assists physicians in the selection of treatments without replacing their own clinical judgement. Various studies have demonstrated that these systems can improve healthcare, reduce inappropriate prescriptions and pharmaceutical costs, monitor antibiotic resistances, and diminish the morbidity and mortality of patients [15,19,20,21].

GERH^®^ is integrated within the routine clinical workflow of our ICU, offering a predictive model that provides timely recommendations [13] and is designed to increase the percentage of patients who receive appropriate empirical antibiotic therapy, as recommended in previous studies [22,23]. According to the present findings, LRM-recommended prescriptions would have been appropriate in terms of the susceptibility of isolated bacteria in 57.6% of lower respiratory tract infection cases, 41.4% of bacteremia cases, and 54.9% of urinary tract infection cases. Higher percentages were published for these infections in the ENVIN study (2018), ranging between 63% and 72% [24]. Nevertheless, the use of LRMs in our ICU would have significantly improved the adequacy of empirical treatment prescriptions in lower respiratory tract infections by 2.2% (*p* = 0.018), although no significant improvement would have been achieved in the cases of bacteremia (3.1%; *p* = 0.070) or urinary tract infection (5.3%; *p* = 0.142). These improvements are modest but similar to previous reports [25,26,27], contributing to evidence that these systems can assist clinical decision-making and improve the adequacy of empirical antibiotic treatments, as previously affirmed [28].

Therapeutic recommendations are provided by LRMs before the responsible microorganism has been defined, and their percentage adequacy is less than when recommendations are made after identifying the etiological agent but before testing its susceptibility [29]. This is the case with PMRTRs, another GERH^®^ instrument, whose prescription recommendations were reported to be appropriate in >82% of cases and to achieve an improvement of 40% in the adequacy of prescriptions for each clinical situation, as we noted in a previous publication [15].

The main study limitation was that it did not consider whether or not physicians had consulted LRMs (available since 2014) before prescribing antibiotics, preventing assessment of the impact of LRM consultations over time on the adequacy of empirical antibiotic therapies. LRMs were designed to inform clinicians about the local epidemiology related to nosocomial infections, allowing them to base empirical antibiotic prescriptions on the likelihood of infection with a specific microorganism and on the accumulated activity of different antibiotics against bacteria isolated in a given focus. The aim was not to replace the judgment of clinicians, which may or may not coincide with LRM recommendations. For this reason, clinicians were not asked to state whether or not their prescription followed these recommendations, thereby preserving their prescribing autonomy. As a novel instrument, an adaptation period can be expected before it is accepted and implemented by physicians, who are also influenced by the perception of resulting improvements in antibiotic prescribing and outcomes.

As currently designed, LRMs do not yield information in relation to other PROA objectives such as the improvement in clinical outcomes and the reduction in antibiotic resistance rates, adverse effects, pharmacological interactions, antibiotic consumption, or pharmaceutical costs. These instruments could be improved by the incorporation of new functionalities that monitor and respond to these objectives and tailor empirical therapy recommendations to the clinical situation of each patient. For instance, prescription decisions could be further supported by integrating data for each patient on clinical observations, laboratory results (biochemistry and microbiology), radiology findings, and/or the concentrations of antibiotics in each tissue sample [30], along with the antibiotic susceptibility data. 

## 5. Conclusions

Although GERH^®^-derived LRMs proved to have a high capacity to predict antibiotic susceptibility and resistance profiles, they produce only a moderate improvement in the adequacy of empirical antibiotic therapy, which is significantly greater in cases of lower respiratory tract infections. According to these findings, LRMs are useful to recommend appropriate prescriptions in approximately 50% of cases but less so in patients with bacteremia or urinary tract infections.

## Figures and Tables

**Table 1:** Percentage concordance between bacterial susceptibility shown by LRMs and susceptibility obtained from antibiograms by year, bacterial group, and antibiotic.

Group/Antibiotic	2007		2008		2009		2010		2011		2012		2013		2014		2015		2016		Mean Concordance 2007–2016
	C	N	C	N	C	N	C	N	C	N	C	N	C	N	C	N	C	N	C	N	
***Enterobacteriaceae***																					
Amikacin	100.0%	55	100.0%	61	90.0%	80	93.5%	62	89.7%	39	100.0%	23	100.0%	24	97.0%	101	95.6%	91	97.6%	82	95.9%
Amoxicillin-clavulanic acid	69.6%	46	69.0%	42	55.7%	70	61.4%	57	64.9%	37	62.7%	75	66.3%	86	76.2%	122	65.9%	123	59.6%	102	65.5%
Aztreonam	78.9%	19	93.3%	30	76.9%	13	100.0%	1	0.0%	1	-	-	50.0%	2	69.0%	119	62.8%	94	54.3%	81	66.7%
Cefepime	80.4%	51	85.2%	61	81.3%	80	66.1%	62	71.8%	39	71.1%	76	62.9%	89	76.0%	121	68.0%	125	63.1%	103	71.7%
Cefotaxime	72.5%	51	83.6%	55	77.0%	74	65.5%	58	73.0%	37	65.3%	75	61.2%	85	76.1%	109	67.5%	117	59.6%	99	69.3%
Ceftazidime	72.7%	55	88.5%	61	80.2%	81	66.1%	62	71.1%	38	69.7%	76	61.8%	89	74.6%	122	66.9%	127	57.1%	105	70.0%
Ceftriaxone	100.0%	1	-	-	-	-	-	-	100.0%	1	-	-	-	-	100.0%	1	-	-	-	-	100.0%
Cefuroxime	0.0%	3	73.7%	38	63.2%	68	44.1%	34	70.6%	34	60.8%	74	62.3%	86	81.1%	106	73.3%	116	63.8%	94	67.3%
Ciprofloxacin/Levofloxacin	80.0%	55	86.7%	60	65.4%	81	69.4%	62	52.5%	40	52.6%	76	70.3%	74	69.1%	123	55.6%	126	47.6%	105	63.6%
Gentamicin	92.6%	54	93.4%	61	78.8%	80	85.2%	61	72.5%	40	57.9%	76	69.7%	89	69.1%	123	61.1%	126	54.8%	104	70.8%
Imipenem	97.9%	48	100.0%	60	96.3%	81	93.5%	62	97.4%	39	94.7%	76	89.9%	89	81.5%	97	92.1%	127	92.3%	104	92.6%
Meropenem	100.0%	33	100.0%	25	100.0%	13	100.0%	2	100.0%	1	-	-	-	-	-	-	80.0%	5	100.0%	5	98.8%
Piperacillin-tazobactam	79.6%	54	88.5%	61	86.7%	30	100.0%	2	100.0%	1	-	-	80.0%	20	62.3%	114	64.8%	125	56.9%	102	69.2%
Tobramycin	96.4%	55	96.7%	61	81.5%	81	83.9%	62	76.9%	39	60.2%	76	65.6%	64	71.1%	121	57.9%	126	52.9%	104	71.2%
Total general	84.7%	580	89.5%	676	78.1%	832	74.6%	587	74.1%	386	67.3%	703	69.5%	797	75.1%	1379	68.8%	1428	63.0%	1190	73.2%
**Non-fermenting Gram-negative bacilli**																					
Amikacin	66.7%	60	85.3%	34	90.0%	40	100.0%	32	97.0%	33	100.0%	10	72.7%	11	42.9%	35	81.5%	54	57.4%	54	76.3%
Cefepime	86.4%	59	70.3%	37	50.0%	40	31.3%	32	53.1%	32	58.2%	29	57.1%	35	31.4%	35	61.1%	54	34.6%	55	54.9%
Ceftazidime	62.3%	61	43.2%	37	50.0%	40	34.4%	32	57.6%	33	62.1%	29	62.1%	37	28.2%	39	68.8%	64	49.3%	67	53.1%
Ciprofloxacin/Levofloxacin	88.3%	60	68.4%	38	76.2%	42	43.2%	37	55.9%	34	86.7%	30	56.8%	37	55.3%	38	76.6%	64	66.7%	66	68.8%
Colistin	93.9%	33	88.0%	25	94.9%	39	96.6%	29	87.5%	32	100.0%	28	97.3%	37	34.5%	29	86.0%	50	95.7%	47	88.3%
Gentamicin	85.0%	60	62.2%	37	67.5%	40	81.3%	32	75.8%	33	86.2%	29	62.2%	37	34.3%	35	38.9%	54	41.8%	55	62.1%
Imipenem	62.3%	61	32.4%	37	47.5%	40	37.5%	32	48.5%	33	58.6%	29	54.1%	37	53.3%	30	52.9%	51	41.2%	51	49.4%
Meropenem	41.1%	56	71.4%	35	52.5%	40	31.0%	29	46.7%	30	85.7%	28	62.2%	37	36.7%	30	54.0%	50	36.0%	50	50.7%
Piperacillin-tazobactam	96.6%	59	90.3%	31	50.0%	40	42.9%	28	31.3%	32	71.4%	28	64.9%	37	20.0%	30	68.6%	51	44.0%	50	60.6%
Tobramycin	95.1%	61	100.0%	37	87.5%	40	96.9%	32	93.9%	33	100.0%	29	81.1%	37	77.1%	35	85.2%	54	58.2%	55	86.2%
Total general	77.9%	610	70.1%	348	66.6%	401	59.4%	315	64.9%	325	79.6%	269	66.7%	342	41.7%	336	67.6%	546	52.4%	550	64.9%
**Positive coagulase staphylococci (*S. aureus*)**																					
Ciprofloxacin/Levofloxacin	57.1%	63	25.0%	28	40.0%	20	27.8%	36	37.5%	24	51.9%	27	66.7%	33	93.5%	31	75.8%	33	70.7%	41	56.2%
Clindamycin	50.8%	63	15.4%	26	57.9%	19	72.2%	36	54.2%	24	51.6%	31	93.9%	33	74.2%	31	90.9%	33	75.6%	41	64.4%
Daptomycin	-	-	-	-	-	-	-	-	-	-	100.0%	4	69.8%	31	100.0%	29	100.0%	32	100.0%	41	93.2%
Erythromycin	60.9%	64	17.9%	28	52.6%	19	58.3%	36	41.7%	24	35.5%	31	66.7%	33	54.8%	31	63.5%	33	73.2%	41	54.7%
Gentamicin	93.8%	64	96.6%	29	100.0%	9	100.0%	36	100.0%	24	96.8%	31	84.8%	33	90.3%	31	90.9%	33	95.1%	41	94.3%
Linezolid	100.0%	50	100.0%	29	100.0%	14	100.0%	37	100.0%	23	100.0%	31	97.0%	33	100.0%	31	100.0%	32	92.7%	41	98.8%
Oxacillin	59.0%	61	20.7%	29	60.0%	20	18.9%	37	37.5%	24	48.4%	31	66.7%	33	90.3%	31	69.7%	33	65.9%	41	54.4%
Rifampicin	100.0%	45	100.0%	23	100.0%	19	97.3%	37	54.2%	24	100.0%	31	96.4%	28	-	-	0.0%	1	-	-	93.3%
Teicoplanin	100.0%	64	100.0%	28	100.0%	20	100.0%	37	95.8%	24	100.0%	31	97.0%	33	100.0%	31	100.0%	32	100.0%	40	99.4%
Tobramycin	57.1%	56	24.1%	29	35.0%	20	72.2%	36	54.2%	24	77.4%	31	89.3%	28	-	-	-	-	-	-	59.8%
Vancomycin	100.0%	63	96.6%	29	100.0%	20	100.0%	37	100.0%	23	100.0%	31	97.0%	33	96.8%	31	100.0%	33	100.0%	41	99.1%
Total general	77.1%	593	59.4%	278	73.7%	190	74.8%	365	66.9%	239	76.8%	310	86.3%	351	88.8%	277	87.5%	295	85.9%	368	78.3%
**Negative coagulase staphylococci**																					
Ciprofloxacin/Levofloxacin	64.3%	42	70.8%	65	67.9%	84	69.4%	72	78.3%	60	56.1%	66	63.5%	74	84.6%	39	82.9%	35	70.8%	24	64.6%
Clindamycin	44.2%	43	49.2%	65	64.3%	84	62.3%	69	65.0%	60	43.5%	69	58.1%	74	65.9%	41	69.4%	36	58.3%	24	60.2%
Daptomycin	-	-	-	-	-	-	-	-	-	-	100.0%	33	100.0%	72	100.0%	40	100.0%	36	100.0%	21	99.7%
Erythromycin	79.1%	43	84.4%	64	76.2%	84	72.2%	72	79.7%	59	84.1%	69	91.9%	74	70.7%	41	80.5%	36	91.7%	24	71.0%
Gentamicin	58.1%	43	52.3%	65	42.9%	84	52.8%	72	56.7%	60	40.6%	69	48.0%	73	63.4%	41	69.4%	36	62.5%	24	68.1%
Linezolid	100.0%	35	91.5%	59	93.8%	65	100.0%	65	98.2%	56	89.4%	66	87.5%	64	76.3%	38	69.4%	36	54.6%	22	92.9%
Oxacillin	71.4%	42	84.6%	65	75.0%	84	79.7%	74	80.0%	60	79.7%	69	79.5%	73	92.7%	41	88.9%	36	95.8%	24	71.9%
Rifampicin	87.5%	24	90.2%	51	96.4%	84	94.6%	74	53.3%	60	98.6%	69	100.0%	71	87.5%	8	-	-	100.0%	2	90.9%
Teicoplanin	86.0%	43	100.0%	65	86.9%	84	91.9%	74	76.7%	60	76.8%	69	81.1%	74	97.6%	41	97.2%	36	100.0%	22	92.2%
Tobramycin	47.6%	42	62.5%	64	56.0%	84	67.1%	73	65.0%	60	49.3%	69	62.0%	71	42.9%	7	-	-	-	-	59.1%
Vancomycin	100.0%	43	100.0%	65	98.8%	84	100.0%	72	95.0%	60	100.0%	69	94.6%	74	100.0%	41	100.0%	36	100.0%	24	98.7%
Total general	72.7%	400	78.2%	628	75.4%	821	78.9%	717	74.6%	595	73.1%	717	78.6%	794	82.8%	378	84.2%	323	81.5%	211	77.6%
***Staphylococcus* spp.**																					
Ciprofloxacin/Levofloxacin	60.0%	105	57.0%	93	62.5%	104	55.6%	108	66.7%	84	54.8%	93	64.5%	107	88.6%	70	79.4%	68	70.8%	65	64.6%
Clindamycin	48.1%	106	39.6%	91	63.1%	103	65.7%	105	61.9%	84	46.0%	100	69.2%	107	69.4%	72	79.7%	69	69.2%	65	60.2%
Daptomycin	-	-	-	-	-	-	-	-	-	-	100.0%	37	99.0%	103	100.0%	69	100.0%	68	100.0%	62	99.7%
Erythromycin	68.2%	107	64.1%	92	71.8%	103	67.6%	108	68.7%	83	69.0%	100	84.1%	107	63.9%	72	72.5%	69	80.0%	65	71.0%
Gentamicin	79.4%	107	66.0%	94	53.4%	103	68.5%	108	69.0%	84	58.0%	100	59.4%	106	75.0%	72	79.7%	69	83.1%	65	68.1%
Linezolid	100.0%	85	94.3%	88	94.9%	79	100.0%	102	98.7%	79	92.8%	97	90.7%	97	87.0%	69	83.8%	68	79.4%	63	92.9%
Oxacillin	64.1%	103	64.9%	94	72.1%	104	59.5%	111	67.9%	84	70.0%	100	83.3%	96	91.7%	72	79.7%	69	76.9%	65	71.9%
Rifampicin	95.7%	69	93.2%	74	97.1%	103	95.5%	111	53.6%	84	99.0%	100	99.0%	99	87.5%	8	0.0%	1	100.0%	2	90.9%
Teicoplanin	94.4%	107	100.0%	93	89.4%	104	94.6%	111	82.1%	84	84.0%	100	86.0%	107	98.6%	72	98.5%	68	100.0%	62	92.2%
Tobramycin	53.1%	98	50.5%	93	51.9%	104	68.8%	109	61.9%	84	58.0%	100	69.7%	99	42.9%	7	-	-	-	-	59.1%
Vancomycin	100.0%	106	98.9%	94	99.0%	104	100.0%	109	95.2%	84	100.0%	100	95.3%	107	98.6%	72	100.0%	69	100.0%	65	98.7%
Total general	75.3%	993	72.4%	906	75.1%	1011	77.5%	1082	72.4%	834	74.2%	1027	81.0%	1148	85.3%	655	85.8%	618	84.3%	579	77.6%
***Streptococcus pneumoniae***																					
Cefotaxime	85.7%	7	100.0%	7	100.0%	12	100.0%	6	100.0%	3	100.0%	3	100.0%	8	100.0%	4	100.0%	4	100.0%	7	98.4%
Levofloxacin	87.5%	8	80.0%	5	100.0%	12	100.0%	6	100.0%	3	50.0%	2	90.0%	10	100.0%	4	100.0%	4	100.0%	7	93.4%
Linezolid	-	-	-	-	-	-	100.0%	1	100.0%	2	-	-	100.0%	5	-	-	100.0%	1	100.0%	7	100.0%
Penicillin	42.9%	7	28.6%	7	41.7%	12	60.0%	5	33.3%	3	100.0%	3	50.0%	10	0.0%	4	100.0%	1	100.0%	6	50.0%
Total general	72.7%	22	68.4%	19	80.6%	36	88.9%	18	81.8%	11	87.5%	8	81.8%	33	66.7%	12	100.0%	10	100.0%	27	82.7%
**Enterococci**																					
Ampicillin	90.0%	10	88.9%	9	71.4%	7	100.0%	5	100.0%	5	100.0%	4	100.0%	11	100.0%	12	93.3%	15	100.0%	11	94.4%
Levofloxacin	30.8%	13	77.8%	9	85.7%	7	0.0%	5	80.0%	5	100.0%	4	63.6%	11	33.3%	12	57.1%	14	36.4%	11	52.7%
Daptomycin	-	-	-	-	-	-	-	-	-	-	-	-	-	-	100.0%	6	100.0%	13	100.0%	8	100.0%
Linezolid	100.0%	12	100.0%	9	100.0%	7	100.0%	5	80.0%	5	100.0%	4	90.0%	10	100.0%	9	53.9%	13	100.0%	8	90.3%
Teicoplanin	100.0%	12	66.7%	9	100.0%	7	100.0%	5	100.0%	4	100.0%	4	100.0%	10	100.0%	9	100.0%	13	100.0%	8	96.3%
Vancomycin	100.0%	11	100.0%	9	100.0%	7	100.0%	5	100.0%	5	100.0%	4	100.0%	11	100.0%	12	93.3%	15	100.0%	11	98.9%
Total general	82.8%	58	86.7%	45	91.4%	35	80.0%	25	91.7%	24	100.0%	20	90.6%	53	78.3%	60	83.1%	83	87.7%	57	85.9%
***Haemophilus* spp.**																					
Amoxicillin-clavulanic acid	90.0%	10	92.3%	13	80.0%	5	90.0%	10	100.0%	4	100.0%	13	100.0%	12	100.0%	8	100.0%	8	100.0%	8	95.6%
Ampicillin	30.0%	10	30.8%	13	60.0%	5	80.0%	10	75.0%	4	53.8%	13	58.3%	12	12.5%	8	50.0%	8	25.0%	8	46.1%
Cefotaxime	100.0%	10	100.0%	13	100.0%	5	100.0%	10	100.0%	4	100.0%	13	100.0%	12	100.0%	8	100.0%	8	100.0%	8	100.0%
Ciprofloxacin/Levofloxacin	100.0%	10	100.0%	13	100.0%	5	100.0%	10	100.0%	4	100.0%	13	100.0%	12	100.0%	8	100.0%	8	100.0%	8	100.0%
Erythromycin	90.0%	10	76.9%	13	80.0%	5	50.0%	10	100.0%	4	15.3%	13	90.9%	11	75.0%	8	62.5%	8	87.5%	8	68.9%
Total general	82.0%	50	80.0%	65	84.0%	25	84.0%	50	95.0%	20	73.8%	65	89.8%	59	77.5%	40	82.5%	40	82.5%	40	82.1%
**All bacteria**																					
Amikacin	82.6%	115	94.7%	95	90.0%	120	95.7%	94	93.1%	72	100.0%	33	91.4%	35	83.1%	136	90.3%	145	81.6%	136	88.7%
Amoxicillin-clavulanic acid	73.2%	56	74.5%	55	57.3%	75	65.7%	67	68.3%	41	68.2%	88	70.4%	98	77.7%	130	67.9%	131	62.7%	110	68.7%
Ampicillin	60.0%	20	54.5%	22	66.7%	12	86.7%	15	88.9%	9	64.7%	17	78.3%	23	65.0%	20	78.3%	23	68.4%	19	70.0%
Aztreonam	78.9%	19	93.3%	30	76.9%	13	100.0%	1	0.0%	1	-	-	100.0%	1	69.1%	97	62.8%	94	54.3%	81	66.8%
Cefepime	83.6%	110	79.6%	98	70.8%	120	54.3%	94	63.4%	71	67.6%	105	61.3%	124	66.0%	156	65.9%	179	53.2%	158	66.1%
Cefotaxime	77.9%	68	88.0%	75	81.3%	91	73.0%	74	77.3%	44	71.4%	91	68.6%	105	78.5%	121	70.5%	129	64.9%	114	74.3%
Ceftazidime	67.2%	116	71.4%	98	70.2%	121	55.3%	94	64.8%	71	67.6%	105	61.9%	126	63.4%	161	67.5%	191	54.1%	172	64.1%
Ceftriaxone	100.0%	1	-	-	-	-	-	-	100.0%	1	-	-	-	-	100.0%	1	-	-	-	-	100.0%
Cefuroxime	0.0%	3	73.7%	38	63.2%	68	44.1%	34	70.6%	34	60.8%	74	66.3%	86	81.1%	106	73.3%	116	63.8%	94	67.8%
Ciprofloxacin/levofloxacin	72.1%	251	71.1%	218	68.9%	251	59.2%	228	62.9%	170	61.9%	218	67.7%	251	72.2%	255	68.0%	284	60.7%	262	66.7%
Clindamycin	48.1%	106	39.6%	91	63.1%	103	65.7%	105	61.9%	84	46.0%	100	69.2%	107	69.4%	72	79.7%	69	69.2%	65	60.2%
Colistin	93.9%	33	88.0%	25	94.9%	39	96.6%	29	87.5%	32	100.0%	28	97.3%	37	34.5%	29	86.0%	50	95.7%	47	88.3%
Daptomycin	-	-	-	-	-	-	-	-	-	-	100.0%	37	99.0%	103	100.0%	75	100.0%	81	100.0%	70	99.7%
Erythromycin	70.1%	117	65.7%	105	72.2%	108	66.1%	118	70.1%	87	62.8%	113	84.7%	118	65.0%	80	71.4%	77	80.8%	73	70.8%
Gentamicin	84.2%	221	74.0%	192	65.0%	223	75.6%	201	71.3%	157	61.9%	205	63.8%	232	65.7%	230	61.5%	249	59.8%	224	68.0%
Imipenem	78.0%	109	74.2%	97	80.2%	121	74.5%	94	75.0%	72	84.8%	105	79.4%	126	75.8%	149	80.9%	178	75.5%	155	78.0%
Linezolid	100.0%	97	94.8%	97	95.3%	86	100.0%	108	97.7%	86	93.1%	101	91.1%	112	82.1%	78	79.3%	82	83.9%	78	92.3%
Meropenem	62.9%	89	83.3%	60	64.2%	53	35.5%	31	48.4%	31	85.7%	28	62.2%	37	36.7%	30	56.4%	55	41.8%	55	59.3%
Oxacillin	64.1%	103	64.9%	94	72.1%	104	59.5%	111	67.9%	84	70.0%	100	75.5%	106	91.7%	72	79.7%	69	76.9%	65	71.2%
Penicillin	42.9%	7	28.6%	7	41.7%	12	60.0%	5	33.3%	3	100.0%	3	50.0%	10	0.0%	4	100.0%	1	100.0%	6	50.0%
Piperacillin-tazobactam	88.5%	113	89.1%	92	65.7%	70	46.7%	30	33.3%	33	71.4%	28	70.2%	57	53.5%	144	65.9%	176	52.6%	152	65.5%
Rifampicin	95.7%	69	93.2%	74	97.1%	103	95.5%	111	53.6%	84	99.0%	100	99.0%	99	87.5%	8	0.0%	1	100.0%	2	90.9%
Teicoplanin	95.0%	119	97.1%	102	90.1%	111	94.8%	116	83.0%	88	84.6%	104	87.2%	117	98.8%	81	98.8%	81	100.0%	70	92.5%
Tobramycin	76.2%	214	74.9%	191	68.9%	225	77.8%	203	72.4%	156	64.9%	205	70.5%	200	71.2%	163	66.1%	180	54.7%	159	70.0%
Vancomycin	100.0%	117	99.0%	103	99.1%	111	100.0%	114	95.5%	89	100.0%	104	95.8%	118	98.8%	84	98.8%	84	100.0%	76	98.7%
Total general	78.5%	2273	78.1%	2059	75.1%	2340	74.2%	2077	71.9%	1600	72.8%	2092	75.6%	2429	73.3%	2482	73.2%	2725	67.0%	2443	73.9%

***C***, the percentage of concordance in the assessment of antibiotic susceptibility of each bacterium between the information provided by LRMs and that obtained in the in vitro susceptibility study; ***N****,* for each year, the number of times in which each antibiotic was tested against bacteria of this group (number of trials with bacterial susceptibility against this antibiotic and comparison with the information provided by LRMs).

**Table 2:** Percentage of concordance between bacterial susceptibility depicted by LRMs and that obtained from antibiograms by year and type of infection.

**Type of infection**	**2007**			**2008**			**2009**			**2010**			**2011**			**2012**		
	**C**	**N**	**M**	**C**	**N**	**M**	**C**	**N**	**M**	**C**	**N**	**M**	**C**	**N**	**M**	**C**	**N**	**M**
**Respiratory**	80.0%	1568	173	79.4%	1081	117	75.5%	1185	130	72.1%	1076	123	70.8%	768	83	74.4%	1030	118
**Bacteremia**	74.4%	598	70	75.8%	810	87	75.7%	904	95	77.6%	843	90	75.6%	697	71	74.7%	883	88
**Urinary**	79.4%	107	13	81.5%	168	17	71.3%	251	25	70.9%	158	17	59.3%	135	13	54.7%	179	20
**All**	78.5%	2273	256	78.1%	2059	221	75.1%	2340	250	74.2%	2077	230	71.9%	1600	167	72.8%	2092	226
**Type of infection**	**2013**			**2014**			**2015**			**2016**			**Mean concordance** **2007–2016**					
	**C**	**N**	**M**	**C**	**N**	**M**	**C**	**N**	**M**	**C**	**N**	**M**	**C**	**N**	**M**			
**Respiratory**	74.4%	1217	142	70.6%	1652	186	72.4%	1543	159	66.5%	1618	174	73.5%	12738	1405			
**Bacteremia**	80.8%	943	92	77.9%	672	82	76.2%	728	76	68.9%	491	52	76.1%	7569	803			
**Urinary**	63.2%	269	32	81.6%	158	20	70.9%	454	49	66.2%	334	37	69.3%	2213	243			
**All**	75.6%	2429	266	73.3%	2482	288	73.2%	2725	284	67.0%	2443	263	74.0%	22520	2451			

***C***, the percentage agreement on the antibiotic susceptibility of each bacteria between LRMs and in vitro susceptibility studies; ***N***, for each year, the total number of susceptibility studies of bacteria isolated in this type of sample versus the group of antibiotics (number of tests with results for bacterial susceptibility to the antibiotic and comparison with information provided by LRMs); ***M***, for each year, the total number of bacteria isolated in patients with infections of each type.

**Table 3:** Percentage adequacy of the empirical prescription of an antibiotic if based on LRM recommendations with reference to the actual susceptibility of isolated bacteria.

Antibiotics	Clinical Category According to in Vitro Susceptibility Test	Clinical Category Defined by LRMs		Total	Percentage Adequacy
		S	R		
Amikacin	S	854	50	904	87.1%
	R	61	16	77	
Amoxicillin-clavulanic	S	251	199	450	29.5%
	R	67	334	401	
Ampicillin	S	108	37	145	60.0%
	R	17	18	35	
Aztreonam	S	133	82	215	39.3%
	R	31	92	123	
Cefepime	S	604	281	885	49.7%
	R	131	199	330	
Cefotaxime	S	538	167	705	59.0%
	R	67	140	207	
Ceftazidime	S	574	311	885	45.7%
	R	140	230	370	
Cefuroxime	S	209	156	365	32.0%
	R	54	234	288	
Ciprofloxacin/levofloxacin	S	889	612	1501	37.2%
	R	184	703	887	
Clindamycin	S	195	309	504	21.6%
	R	50	348	398	
Colistin	S	306	18	324	87.7%
	R	23	2	25	
Daptomycin	S	365	1	366	99.7%
	R	0	0	0	
Erythromycin	S	116	243	359	11.6%
	R	48	589	637	
Gentamicin	S	976	540	1516	45.7%
	R	144	474	618	
Imipenem	S	801	197	998	66.4%
	R	68	140	208	
Linezolid	S	851	43	894	92.0%
	R	29	2	31	
Meropenem	S	201	144	345	42.9%
	R	47	77	124	
Oxacillin	S	133	217	350	14.6%
	R	45	513	558	
Piperacillin-tazobactam	S	436	226	662	48.7%
	R	83	150	233	
Rifampicin	S	376	7	383	57.8%
	R	53	215	268	
Teicoplanin	S	912	29	941	92.2%
	R	45	3	48	
Tobramycin	S	851	435	1286	44.9%
	R	133	477	610	
Vancomycin	S	987	6	993	98.7%
	R	7	0	7	

**S**, susceptible clinical category; **R**, resistant clinical category.

**Table 4:** Percentage adequacy of the empirical prescription of an antibiotic if based on LRM recommendations with reference to the actual susceptibility of isolated bacteria by year and by type of infection.

Type of Infection	2007	2008	2009	2010	2011	2012	2013	2014	2015	2016	2007–2016
**Respiratory**	58.4%	73.1%	62.2%	63.8%	55.5%	65.7%	60.1%	47.3%	50.0%	50.9%	57.6%
**Bacteremia**	41.1%	41.7%	39.5%	41.8%	39.7%	37.5%	40.1%	46.0%	49.7%	38.1%	41.4%
**Urinary**	78.5%	78.0%	59.4%	65.2%	48.9%	46.9%	43.1%	57.0%	51.0%	47.9%	54.9%
**All**	54.8%	61.1%	53.1%	55.0%	48.1%	52.3%	50.5%	47.5%	50.1%	47.9%	51.9%

**Table 5:** Percentage adequacy of empirical antibiotic treatment by year and by type of infection.

**Type of Infection**	**2007**			**2008**			**2009**			**2010**			**2011**			**2012**		
	**% adequacy**	***N***	***M***	**% adequacy**	***N***	***M***	**% adequacy**	***N***	***M***	**% adequacy**	***N***	***M***	**% adequacy**	***N***	***M***	**% adequacy**	***N***	***M***
**Respiratory**	47.7%	132	53	65.9%	82	46	52.5%	118	51	60.2%	161	83	49.3%	134	59	58.1%	105	57
**Bacteremia**	46.9%	49	18	40.0%	120	47	38.0%	92	42	34.9%	126	58	32.0%	125	51	50.5%	103	54
**Urinary**	42.9%	7	5	44.4%	9	6	16.7%	6	2	61.5%	13	7	22.2%	9	4	53.8%	13	9
**All**	47.3%	188	76	50.2%	211	99	45.4%	216	95	49.7%	300	148	40.3%	268	114	54.3%	221	120
**Type of Infection**	**2013**			**2014**			**2015**			**2016**			**Adequacy 2007–2016**					
	**% adequacy**	***N***	***M***	**% adequacy**	***N***	***M***	**% adequacy**	***N***	***M***	**% adequacy**	***N***	***M***	**% adequacy**	***N***	***M***			
**Respiratory**	62.4%	93	60	48.8%	125	61	63.6%	132	89	49.5%	107	61	55.4%	1189	620			
**Bacteremia**	32.3%	96	46	37.9%	58	29	40.0%	75	42	35.4%	48	24	38.3%	892	411			
**Urinary**	62.5%	16	10	71.4%	7	4	43.3%	30	18	66.7%	9	7	49.6%	119	72			
**All**	48.3%	205	116	46.3%	190	103	53.6%	237	149	46.3%	164	92	48.2%	2200	1112			

***N***, for each year, the total number of comparisons between empirically prescribed antibiotics and the result of in vitro susceptibility tests for each bacterium isolated in ICU patients; ***M***, for each year, the total number of bacteria isolated whose in vitro susceptibility study was compared to the empirical antibiotic treatment to calculate the percentage adequacy.
